# Optimizing Gantry Breakpoint Angles in Proton Therapy: Enhancing Efficiency and Patient Experience

**DOI:** 10.1016/j.ijpt.2024.03.001

**Published:** 2024-04-20

**Authors:** Xueyan Tang, Amanda J. Deisher, Daniel W. Mundy, Jon J. Kruse, Anita Mahajan, Jing Qian, Jedediah E. Johnson

**Affiliations:** Department of Radiation Oncology, Mayo Clinic, Rochester, MN, USA

**Keywords:** Proton therapy, Gantry breakpoint, Patient setup, Treatment efficiency, Workflow optimization

## Abstract

**Purpose:**

The breakpoint for a 360° radiotherapy gantry is typically positioned at 180°. This arbitrary setting has not been systematically evaluated for efficiency and may cause redundant gantry rotation and extended setup times. Our study aimed to identify an optimal gantry breakpoint angle for a full-gantry proton therapy system, with the goal of minimizing gantry movement.

**Materials and Methods:**

We analyzed 70 months of clinically delivered proton therapy plans (9152 plans, 131 883 fractions), categorizing them by treatment site and mapping the fields from a partial-gantry to full-gantry orientation. For each delivered fraction, we computed the minimum total gantry rotation angle as a function of gantry breakpoint position, which was varied between 0° and 360° in 1° steps. This analysis was performed separately within the entire plan cohort and individual treatment sites, both with and without the capability of over-rotating 10° past the breakpoint from either direction (20° overlap). The optimal gantry breakpoint was identified as one which resulted in a low average gantry rotation per fraction.

**Results:**

Considering mechanical constraints, 130° was identified as a reasonable balance between increased gantry-rotation efficiency and practical treatment considerations. With a 20° overlap, this selection reduced the average gantry rotation by 41.4° per fraction when compared to the standard 180° breakpoint. Disease site subgroups showed the following reduction in average gantry rotation: gastrointestinal 192.2°, thoracic 56.3°, pediatric 44.9°, genitourinary 19.9°, central nervous system 10.7°, breast 2.8°, and head and neck 0.1°.

**Conclusion:**

For a full-gantry system, a breakpoint of 130° generally outperforms the conventional 180° breakpoint. This reduction is particularly impactful for gastrointestinal, pediatric, and thoracic sites, which constitute a significant proportion of cases at our center. The adjusted breakpoint could potentially streamline patient delivery, alleviate mechanical wear, and enhance treatment precision by reducing the likelihood of patient movement during delivery.

## Introduction

In contemporary proton therapy, the efficacy and accuracy of cancer treatment rely heavily on gantry systems. These systems come in 2 primary configurations: the partial gantry and the full gantry. The partial-gantry is designed to rotate through a semicircular path of 190° to 220°,^1^ while the full-gantry offers a complete circular range of 360°.^2^ This distinction is crucial as it directly influences the versatility of treatment delivery. The full-gantry system incorporates a feature known as the gantry breakpoint—a predefined angle where the gantry rotation ceases and reverses to prevent mechanical damage, such as cable twisting. To improve treatment efficiency, some manufacturers have introduced gantry rotation overlap, where the gantry is allowed to rotate slightly past its breakpoint before reversing direction.

Beam arrangements in proton therapy are carefully chosen to maximize tumor dose while sparing healthy tissue, with consideration of biological dose enhancement and potential range uncertainties.^3^, ^4^ These arrangements almost always necessitate 1 or more gantry rotations per treatment plan. Historically, the gantry breakpoint has been standardized to 180°, a choice that has not been critically evaluated for its effectiveness. Certain therapeutic plans, such as those involving 2 posterior oblique beams—a common scenario in proton therapy—result in the gantry approaching a full rotation between fields. Mechanical limitations and patient safety considerations usually limit the gantry’s rotational speed to 6° per second, implying that a full 360° rotation could take more than a minute when accounting for acceleration and deceleration.

Optimizing the gantry breakpoint position could potentially refine treatment workflows. Through a comprehensive analysis of how the total gantry rotation per delivered fraction is affected by varying breakpoint angles, we aim to pinpoint a more effective breakpoint position. A well-optimized breakpoint could potentially shorten treatment times, resulting in reduced patient discomfort and enhanced operational efficiency of treatment facilities.^5^ The rationale for this optimization is not merely operational but also deeply rooted in the clinical imperative to deliver patient-centric care with heightened efficiency and precision.

## Materials and methods

### Data compilation

**Figure 1:**
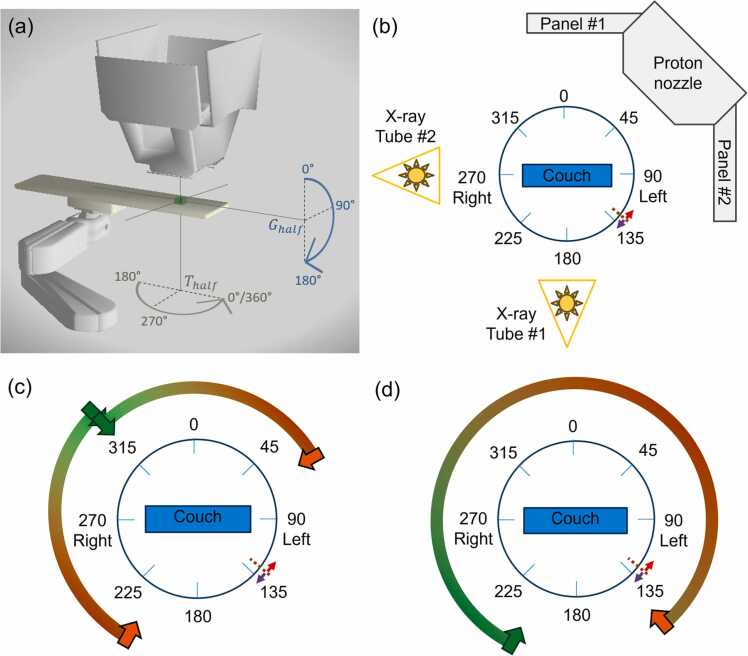
(a) Couch and gantry geometry of the half-gantry system. The green box marks the approximate location of a patient’s head in the head-first supine position. The blue arrow shows the direction of the gantry rotation, and the gray arrow shows the direction of the couch rotation. Plots were made using the Collision Avoider software.^1^ (b) View of a full gantry positioned at *G*_full_ = 45° from the perspective of the patient’s feet (head-first supine), showing 2 x-ray tubes opposite the imaging panels on the gantry. The drawing shows a gantry breakpoint at 130° with 20° of overlap, corresponding to 120° to 140°. (c) Two small field-of-view cone-beam CT rotation paths that start at 315° and cover a 110° arc: 315° to 205° (counterclockwise) and 315° to 65° (clockwise). (d) Large field-of-view cone-beam CT rotation path starting at 210° and rotating 290° through the breakpoint to 140° (clockwise). CT, Computed Tomography.

From January 2018 through October 2023, we delivered 9152 unique proton therapy plans using the Hitachi Probeat-V Proton Beam Therapy System, which has a half-gantry (190°) configuration. We excluded data from our first 2.5 years of operation (June 2015-December 2017) so that the analyzed data represented our mature planning approach. We queried our ARIA radiation oncology information system (Varian Medical Systems, Inc) for the gantry and treatment couch angles of each delivered proton field. The plans were broadly categorized as breast, central nervous system (CNS), gastrointestinal (GI), genitourinary (GU), head and neck (H&N), pediatric, and thoracic, with the remainder classified as “other.” For this analysis, the gantry angle and couch angle delivered with the half-gantry system (*G*_half_ and *T*_half_, respectively) were mapped to a corresponding full-gantry angle, *G*_full_. The geometry of the gantry and the couch in the full-gantry system as well as the half-gantry system is shown in Figure 1. For left-sided fields in the head-first supine patient orientation, encompassing table angles *T*_half_ between 270° and 360°, the gantry angle was unchanged (*G*_full_ = *G*_half_). Right-sided fields, for which *T*_half_ falls inside 180° and 270°, required a mirroring of the gantry around the vertical axis (*G*_full_ = 360°−*G*_half_). For fields where the half-gantry table angle is 270°, the half-gantry rotation is in the sagittal plane of the patient. To recreate these “vertex” fields, the full-gantry configuration requires the table to rotate 90° left or right into the gantry bore. While this capability is not available in all commercial proton systems, some systems do allow for vertex field orientations by utilizing shorter treatment tables. Assuming a left-sided breakpoint, we choose to orient the patient’s feet toward *G*_full_ = 90° so that the potential breakpoint is opposite the vertex field and the mirroring gantry mapping applies. In conclusion, the rules for mapping the gantry angle from a half-gantry system to a full-gantry system can be summarized below:

$$G_{\mathrm{full}}=\left\{\begin{array}{c} 360{}^{\circ}-G_{\mathrm{half}}\;\mathrm{if}\;T_{\mathrm{half}}\in [180{}^{\circ},270{}^{\circ}]\\ G_{\mathrm{half}}\;\mathrm{if}\;T_{\mathrm{half}}\in (270{}^{\circ},360{}^{\circ}] \end{array}\right)$$

### Determination of optimal gantry breakpoint angle

To determine the gantry breakpoint angle that minimizes total gantry rotation per patient, each delivered fraction was assessed by adjusting the gantry breakpoint from 0° to 360° in 1° increments. By focusing our analysis on the average gantry rotation per delivered fraction instead of per plan, we effectively weighted the assessment to reflect the actual usage of the treatment machine, recognizing that each fraction contributes uniquely to the cumulative operational burden and machine utilization. Since a proton therapy plan usually includes multiple beams, for every breakpoint angle, we computed the total gantry rotation for each possible sequence of beam delivery; the smallest resulting value defined the minimal total gantry rotation at that breakpoint. Such analysis was performed on all the delivered fractions, considering both scenarios with and without a 20° gantry rotation overlap (±10° from the breakpoint). The minimal total gantry rotation per treated fraction was first averaged over all fractions and then charted against the varying gantry breakpoints. Subsequently, the same analysis was performed for treatment-site-specific groups in order to isolate the impact for different cohorts of patients.

### Challenges of modeling noncoplanar fields

There are challenges in this analysis with respect to the handling of “noncoplanar” fields, which we define as including couch rotations of >30° off of a true axial/coplanar geometry. The minimum gantry rotation required per fraction for a given breakpoint was determined by ordering the fields by gantry angle, without considering couch angle. The fields were arranged without prioritizing coplanar fields first and segregating vertex fields. The minimum gantry rotation arc might not be feasible in practice when considering patient clearance, though this simplification is not thought to give an advantage to either breakpoint. To ensure that our assumptions for handling the complexities of noncoplanar fields did not distort our overall conclusions, we performed a subset analysis in which we removed fractions with noncoplanar fields.

### Consideration of patient setup and imaging workflow

After an alternative to a 180° breakpoint was identified, the patient setup and imaging workflows were evaluated to determine if the proposed breakpoint would lengthen or complicate patient setup, negating any potential gains in efficiency for rotation between treatment beams. The workflow considered will (1) move the patient from outside the gantry to isocenter, (2) acquire an orthogonal kV x-ray pair, and (3) acquire a cone-beam computed tomography (CBCT) before starting treatment fields.

## Results

The minimum total gantry rotation as a function of breakpoint was computed for the entire set of 131 833 delivered fractions. Comprehensive data for individual treatment sites, such as the number of plans, fractions, and fields delivered for each treatment site, are detailed in Table 1.

**Table 1:** Statistics of the delivered treatments and gantry rotation data for individual treatment sites and the whole cohort.

Treatment sites		Breast	CNS	GU	H&N	GI	Pediatric	Thoracic	Other	Whole cohort
# Plans		1635	985	1694	1346	753	1066	603	1070	9152
# Fractions		22 017	21 501	13 236	25 187	12 982	12 825	8460	15 675	131 883
# Fields		56 005	68 755	40 309	86 473	30 263	39 672	22 621	44 302	388 400
Avg gantry rot (deg)	180° Breakpoint	90.5	199.7	175.2	214.2	274.1	213.3	203	N/A	192.9
	130° Breakpoint	87.7	189	155.3	214.1	81.9	168.4	145.7	N/A	151.5
Avg rot reduction (deg)		2.8	10.7	19.9	0.1	192.2	44.9	56.3	N/A	41.4

**Abbreviations:** CNS, central nervous system; GI, gastrointestinal; GU, genitourinary; H&N, head and neck.

### Analysis of the whole cohort

**Figure 2:**
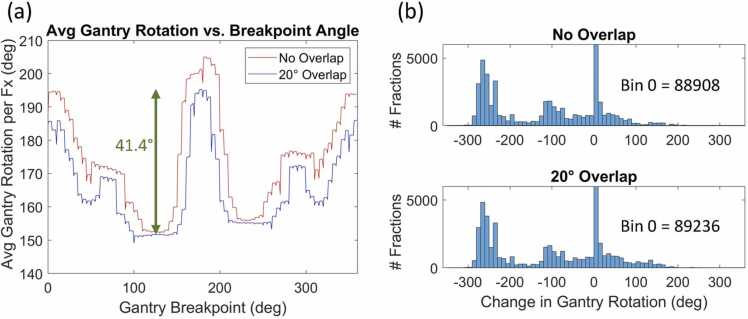
(a) The relationship between the average gantry rotation per fraction and the gantry breakpoint location for the whole cohort. (b) Histograms of the difference in the gantry rotation angle for each fraction between 180° and 130° breakpoints using a bin size of 10°, with and without 20° gantry rotation overlap.

Figure 2a displays the minimum total gantry rotation per fraction versus hypothetical gantry breakpoint angle. The red line shows the values for no gantry rotation overlap, while the blue line represents the scenario with a 20° overlap. Considering mechanical constraints, 130° was identified as a reasonable balance between increased gantry-rotation efficiency and practical treatment considerations. The green arrow indicates the 41.4° change in gantry rotation (with 20° overlap) when comparing the proposed 130° breakpoint to the standard 180° breakpoint. Histograms in Figure 2b chart the distribution of the change in gantry rotation per patient between the 130° and 180° breakpoints using a bin size of 10°, comparing scenarios with and without the 20° gantry rotation overlap. Note that the bin containing 0° includes more than 80 000 entries in both histograms and has been cropped to enhance the visibility of the other bins. For no gantry overlap, the change in breakpoint from 180° to 130° results in <90° of rotation difference for 76.8% of the delivered fractions, increases more than 90° for 1.3%, and decreases more than 90° for 21.9%. For 15.2% of the fractions, the total rotation per fraction decreases by more than 200°. For the 20° overlap, the change is similar, which is <90° of rotation difference for 78.0% of the delivered fractions, increases more than 90° for 1.9%, and decreases more than 90° for 20.1%. The total rotation per fraction decreases by more than 200° for 14.8% of the fractions with the change in breakpoint.

### Subset analysis of coplanar fields

Noncoplanar fields made up 14% of our total delivered fields and were present in 29% of all fractions. The subanalysis of 71% of all fractions, which only included coplanar fields, was qualitatively similar to that of the whole cohort. In particular, the shape of the minimum total gantry rotation per fraction versus hypothetical gantry breakpoint angle is similar to Figure 2. Assuming 20° overlap, the average gantry rotation reduction is 55.8°, confirming the superiority of the 130° breakpoint over the traditional 180° location in this subset (Supplemental Figure). This demonstrates consistency between these multiple analyses and provides assurance that any assumptions made with the treatment of noncoplanar fields have a negligible impact on our overall conclusions.

### Site-specific evaluation

**Figure 3:**
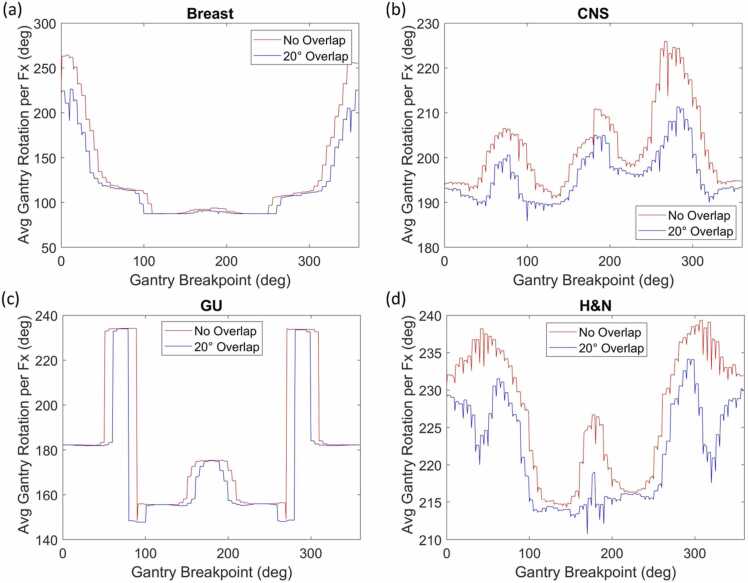
Average gantry rotation per fraction versus breakpoint location for (a) breast, (b) CNS, (c) GU, and (d) H&N plans. Abbreviations: CNS, central nervous system; GU, genitourinary; and H&N, head and neck.

**Figure 4:**
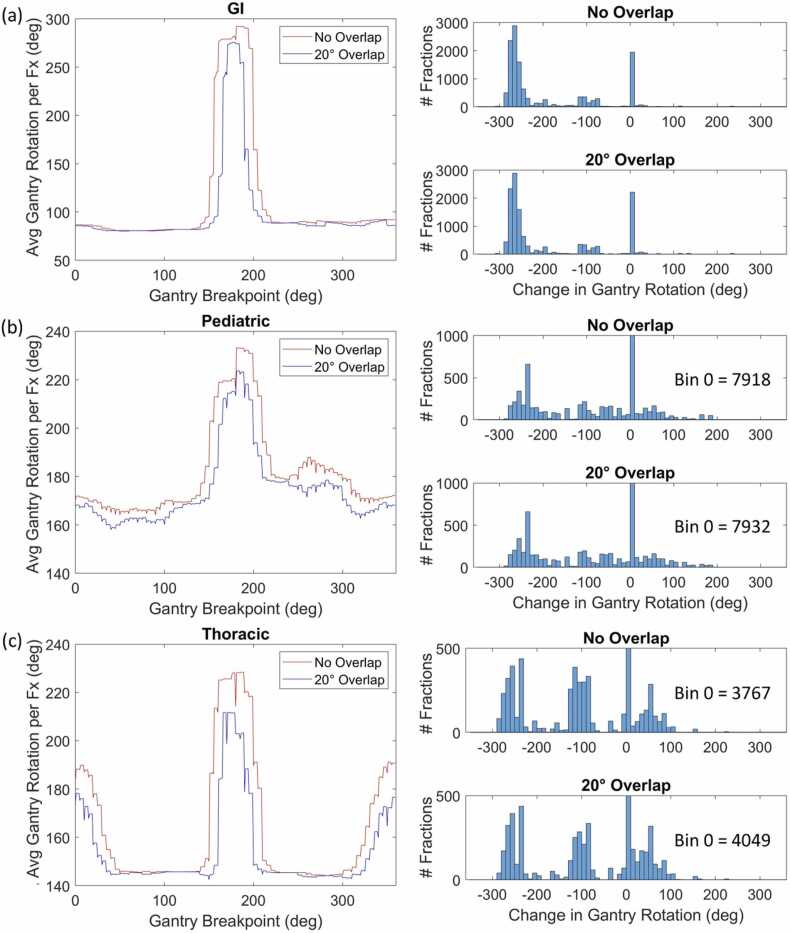
Average gantry rotation per patient versus breakpoint location, accompanied by histograms of gantry rotation differences for (a) GI, (b) pediatric, and (c) thoracic plans. Abbreviations: GI, gastrointestinal.

Figure 3 presents the average gantry rotation per patient for plans divided into breast, CNS, GU, and H&N groupings. These disease sites share the common feature that the variation in gantry rotation angles between the 180° and 130° breakpoints is relatively minor. Figure 4 provides a similar evaluation for GI, pediatric, and thoracic plans, accompanied by histograms showing the difference in gantry rotation angle resulting from a change between 180° and 130° breakpoints using a bin size of 10°, again comparing scenarios with and without the 20° gantry rotation overlap.

Of note, the “pediatric” category includes all pediatric treatment sites aggregated together in 1 group, as the plan labeling scheme in our clinical database did not allow for further programmatic substratification. Although this group contains a heterogeneous mixture of plan types and associated beam angle collections, we want to ensure that gantry breakpoint changes will not negatively affect pediatric patients, as their treatments can be more logistically complex. Average gantry rotation analysis was not performed for the “other” group because these plans represent a collection of diverse sites and their corresponding beam geometries, and as such, there is not clear clinical utility for analyzing this subgroup separately. Note that these plans are still included, however, in the analysis for the “whole cohort” data set.

### Consideration of patient setup and imaging workflow

Given a left-handed robot, access to the patient will be via the patient’s left side, requiring a therapist to walk around the patient’s head to reach the right side. For this reason, the gantry is most likely to be at 0° (neutral, allowing access to both sides of the patient), 315°, or 225° (which carries more risk of colliding with a radiation therapist’s legs during patient setup) as the patient is moved from load position to isocenter. Gantry positions of 315° or 225° would not require gantry rotation to obtain lateral and posterior pairs of 2D images for alignment. For a given patient, the gantry angles during loading and kV pair acquisition would be the same for breakpoints at 180° or 130°.

The key difference in the imaging workflow across the 2 gantry breakpoints is driven by the CBCT process. An ideal CBCT acquisition would end with the gantry in position for the first treatment angle. Dual-panel acquisition of a small field-of-view (FOV) CBCT will require 110° of rotation, with 290° required for a large-FOV CBCT. Figure 1c shows 2 possible small-FOV CBCT acquisitions starting at 315°. Note that both paths for the small-FOV CBCT would be accessible with either a 130° breakpoint or the traditional 180° breakpoint. However, for a large-FOV CBCT, after a kV pair is acquired at a gantry angle of 225°, an additional 15° rotation is required with a 130° breakpoint. While a 180° breakpoint allows large-FOV CBCT acquisition from 225° to 155°, a 130° breakpoint requires an additional 15° rotation to set up for a CBCT acquisition from 210° to 140°, as shown in Figure 1d. As this small rotation could be taken during 2D image matching, it is not anticipated to significantly add to the workflow.

## Discussion

Figure 2a shows that the ideal gantry breakpoint angles for reducing the average gantry rotation per fraction for this cohort fall between 100° and 150° when accounting for overlap. This range narrows to 110° to 140° in the absence of overlap. Given certain mechanical constraints specified by a proton therapy equipment manufacturer, a breakpoint angle of 130° was chosen as the proposed setting. A change in breakpoint position from the standard 180° to 130° reduces the average gantry rotation per fraction by 41.4°, equating to a time savings of approximately 7 seconds per fraction on average. This time savings assumes a consistent gantry rotation speed of 6 degrees per second. In practice, there are clinical scenarios in which slower gantry rotation speeds are utilized to prevent collisions between the gantry and the table or patient. This would imply a slower overall average gantry rotation speed, amplifying the projected benefits of a 130° breakpoint. In other words, these results represent a conservative lower bound of potential overall time savings.

As depicted in Figure 2b, for the majority (67.7%) of the patients, the difference in total gantry rotation between the 180° and 130° breakpoints is negligible (<5°). However, for a distinct and notable subset of patients with posterior oblique fields, this difference can reach up to 300°. Therefore, adopting a 130° breakpoint would yield considerable benefits for this group. It is important to acknowledge, however, that a much smaller percentage of the delivered fractions would see a smaller increase in rotation with a non-180° breakpoint. The imbalance between these groups, which is tilted heavily toward an overall benefit, clearly suggests that modifying the breakpoint is holistically advantageous.

It is important to recognize that these results, including the choice of an improved 130° breakpoint angle and the associated overall gantry rotation reduction, are specific to our institution’s clinical experience. Proton radiotherapy clinics that use different planning techniques, fractionation patterns, and patient disease-site mixtures may therefore see different results. This notwithstanding, we expect that shifting the gantry breakpoint away from 180° enough to accommodate 2 paired posterior oblique fields (without the need to rotate the gantry all the way around the patient) will provide an overall benefit in clinics that use this beam geometry routinely.

To provide more context for this specific study, it is useful to provide a general overview of the beam angles for each group. Breast plans typically use 2 anterior beams, with planning angles optimized for the specific target/tissue geometry. A third beam close to a lateral orientation is sometimes added. The CNS group contains a wide variety of beam angles, some of which are noncoplanar to further optimize the target coverage versus organ-at-risk sparing tradeoff and the distribution of high linear energy transfer protons. GU is dominated by prostate patients, which are usually treated with lateral beams and additional anterior oblique beams for hypofractionated cases. Posterior oblique beams are sometimes incorporated when the target includes superior lymph node volumes. Head and neck planning is dependent on the specific target geometry, but generally anterior, posterior, and lateral beams are used for bilateral disease, while a combination of ipsilateral anterior oblique, lateral, and posterior oblique beams is used for unilateral targets. Pairs of posterior oblique beams are utilized for many of the GI sites, including pancreas, esophagus, and rectal/anal. The exception is liver, which primarily features right-sided beams whose angles depend on the location of the lesion. Because the thoracic group includes targets in all parts of the lungs and mediastinum, the beam angles in this group are diverse, and it is challenging to make broad generalizations. There are, however, many patients whose treatment plans utilize posterior and posterior oblique beams. Finally, as previously indicated, it is also difficult to make beam angle categorizations about the “pediatric” group, as it includes sites from the entire body.

Disease site analysis in Figure 3 reveals that breast, CNS, GU, and H&N plans show minimal benefit from the breakpoint shift. This is expected for plans that contain primarily anterior and lateral beams, as the shortest achievable rotation avoids posterior trajectories.^6^, ^7^, ^8^, ^9^ For the breast treatments in Figure 3a, the least efficient breakpoint would be 0°, as our planning procedure commonly uses both left- and right-sided anterior beams. In comparison, GI plans exhibit a nearly 200° decrease in rotation, corresponding to a 33-second reduction in gantry rotation time (Figure 4a) for posterior fields. It is noteworthy that a left-sided breakpoint of 130° allows for all combinations of right-sided beams without loss of rotational efficiency for liver treatments. Pediatric and thoracic plans also benefit from a change, with an average reduction of 60° or around 10 seconds in rotation time. Treatments in GI, pediatric, and thoracic categories (26.0% of the cohort) will gain the most from the gantry breakpoint modification, which is also supported by Table 1. This improvement is due to the use of 2 posterior oblique beams in these plans, which would otherwise be separated by the 180° breakpoint.^10^, ^11^, ^12^ Adjusting the breakpoint to 130° allows the gantry to swing through a small posterior angle and avoids a nearly full 360° rotation for most of these treatments.

The efficiency of the patient setup and imaging workflows is expected to be similar between breakpoints at 130° and 180°. Based on the dry-run tests on both small-FOV and large-FOV CBCTs with different initial beam angles, for dual-tube acquisition, small-FOV CBCTs, it will be possible to choose an appropriate 2D-imaging gantry angle and then end the acquisition near the first treatment angle. For large-FOV CBCTs, which require closer to a full rotation, the choice of CBCT starting point and first treatment beam is likewise expected to be similar for breakpoints at 130° and 180°.

For the gantry rotation reduction analysis, there was no consideration of potential collision issues with a left-handed couch-positioning robot, which are more likely for vertex fields where 145° ≤ *G*_half_ ≤ 180°. However, there were only 902 fields matching this description in this cohort (<0.25%), so that the impact on the analysis is minimal.

A non-180° breakpoint would be a new paradigm for staff familiar with 180°-breakpoint proton gantries or traditional therapeutic x-ray delivery systems. However, as each new facility brings a steep learning curve for a variety of reasons, the training required for a non-180° breakpoint would only represent a minor addition. Furthermore, this transition could be eased with delivery software that suggests a CBCT starting point and a field order that minimizes overall gantry rotation.

The potential gains of an adjusted gantry breakpoint will also depend on the number of patients treated per room per day. These improvements might not be as impactful for clinics with shared beams across multiple treatment rooms, where gantry rotation times are less critical to overall clinical efficiency. On a patient level, changing the breakpoint can decrease time on the treatment table, resulting in a decreased chance of patient movement and higher precision treatment. On a facility level, the decreased overall gantry rotation could be economically beneficial by decreasing wear on the treatment machine, thus saving money on long-term maintenance costs. For a patient mix that leans more heavily toward posterior field arrangements, where the gantry breakpoint change maximally reduces the time per treatment, enough overall savings may be obtained to allow for an extra daily patient treatment slot. Even if the gains are more modest, however, incremental workflow efficiency gains can still alleviate some of the pressure on radiation therapists to stay on a tight clinical schedule, thus contributing to reducing staff burnout.

## Author contribution

Xueyan Tang: Writing- Original draft, Writing- Review & editing, Methodology, Formal analysis. Amanda J. Deisher: Conceptualization, Methodology, Writing- Original draft, Writing- Review & editing, Formal analysis, Data curation. Daniel W. Mundy: Conceptualization, Methodology, Writing- Review & editing. Jon J. Kruse: Conceptualization, Methodology, Writing- Review & editing. Anita Mahajan: Conceptualization, Methodology, Writing- Review & editing, Resources. Jing Qian: Conceptualization, Methodology, Writing- Review & editing. Jedediah Johnson: Conceptualization, Methodology, Formal analysis, Writing- Original draft, Writing- Review & editing, Data curation, Supervision, Project administration.

## Declaration of Conflicts of Interest

The authors have no conflicts to disclose.

## Data Availability

Individual participant data are not available.

## Appendix A Supplementary material

Supplemental Material, Figure 1 (a) The relationship between the average gantry rotation per fraction and the gantry breakpoint location for the subset of plans containing only fields within 30° of an axial geometry (“coplanar” fields). (b) Histograms of the difference in the gantry rotation angle for each fraction between 180° and 130° breakpoints using a bin size of 10°, with and without 20° gantry rotation overlap.
