# Supplementary figures and images for: Optimizing Gantry Breakpoint Angles in Proton Therapy: Enhancing Efficiency and Patient Experience
Source: Int J Part Ther. 2024 Apr 20;11:100007. doi: 10.1016/j.ijpt.2024.03.001 (PMC11095102; doi:10.1016/j.ijpt.2024.03.001)

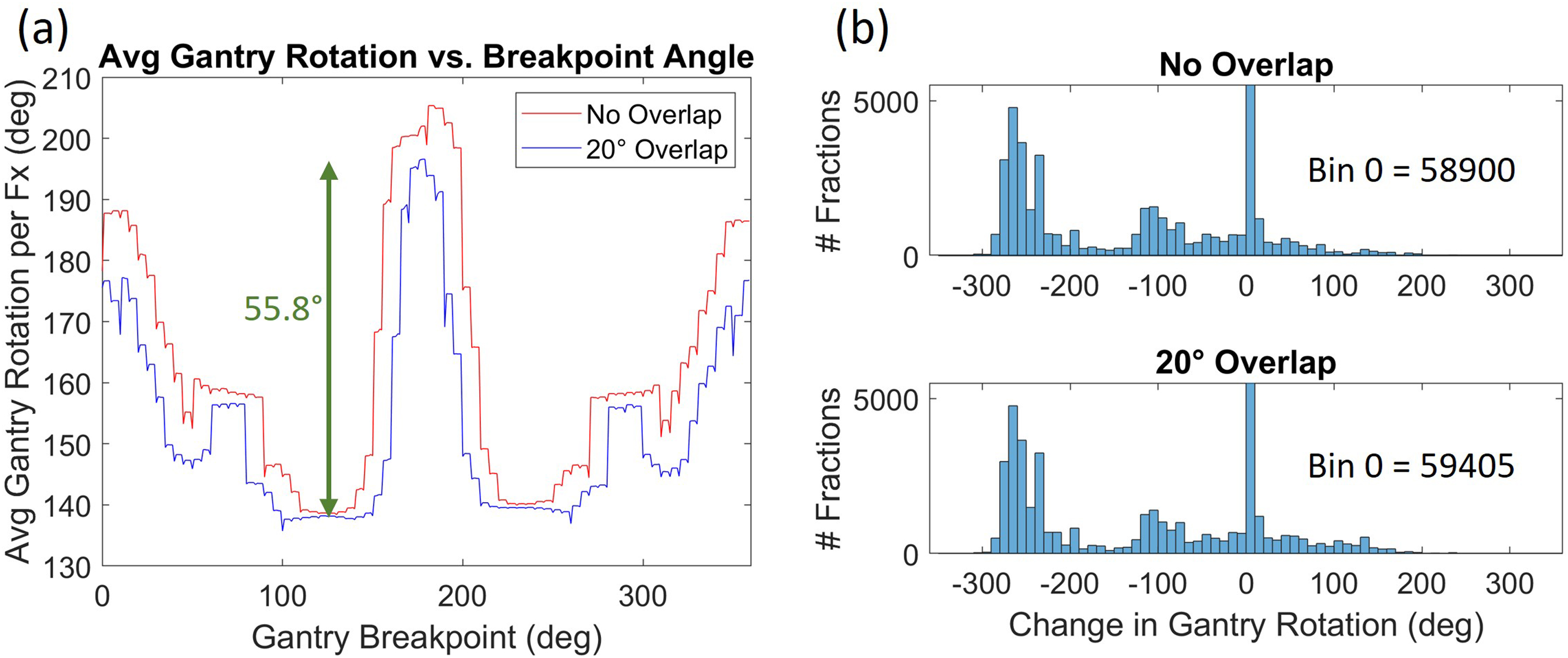

Supplement: Supplemental Material, Figure 1 — (a) The relationship between the average gantry rotation per fraction and the gantry breakpoint location for the subset of plans containing only fields within 30° of an axial geometry (“coplanar” fields). (b) Histograms of the difference in the gantry rotation angle for each fraction between 180° and 130° breakpoints using a bin size of 10°, with and without 20° gantry rotation overlap. [file mmc1.jpg]
